# Supplementary material for: Molecular determinants of avoidance and inhibition of Pseudomonas aeruginosa MexB efflux pump
Source: mBio. 2023 Jul 26;14(4):e01403-23. doi: 10.1128/mbio.01403-23 (PMC10470492; doi:10.1128/mbio.01403-23)

**Figure S1.** Spatial distribution of MexB regions. Left: Architecture of the substrate binding site of MexB with its five main areas highlighted as following: Outer AP-red, Inner AP – green, Interface – blue, DP Groove – gold; DP Cave – cyan. Right: The switch loop is colored in blue and the residues lining the hydrophobic trap are reported as red stick.


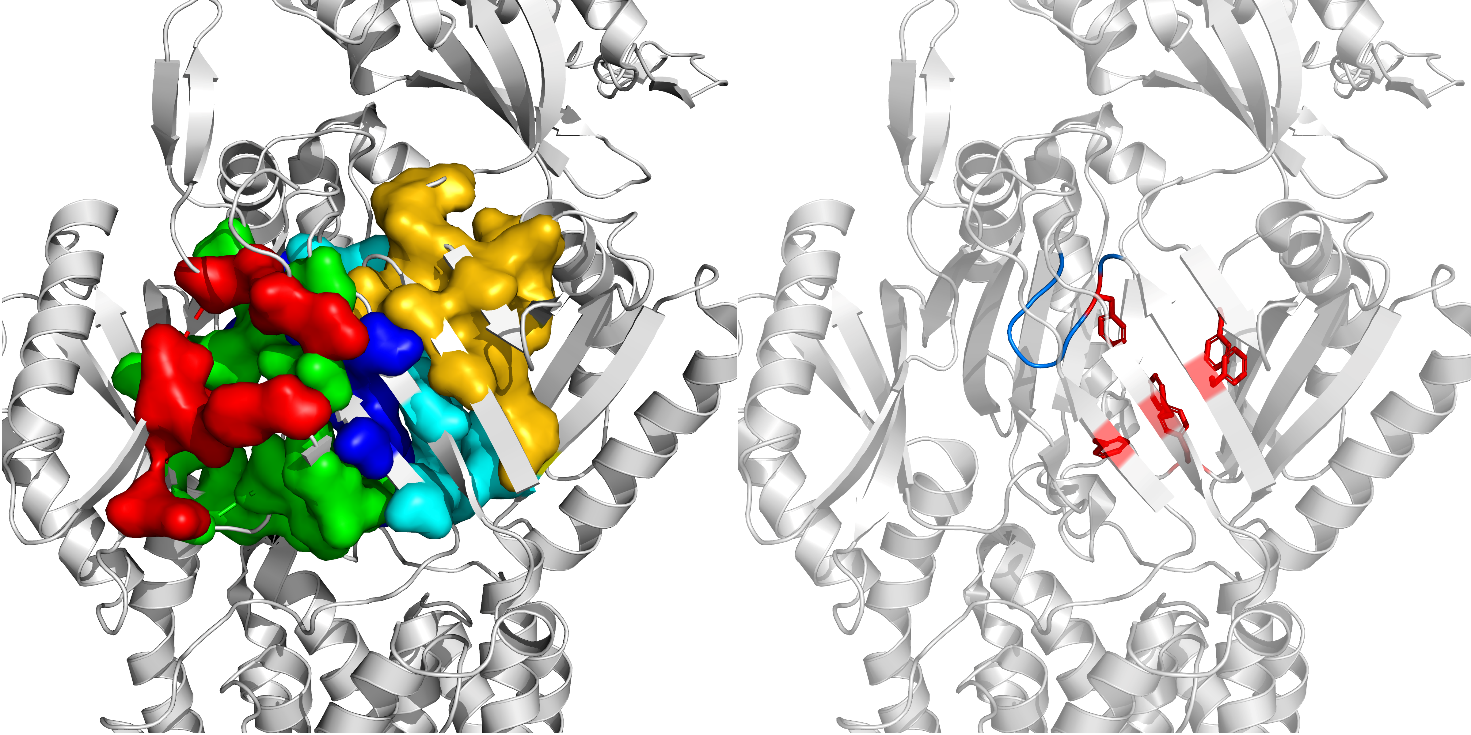

Supplement: Fig. S1 — Spatial distribution of MexB regions. [file mbio.01403-23-s0002.docx]
