# Supplementary material for: Molecular determinants of avoidance and inhibition of Pseudomonas aeruginosa MexB efflux pump
Source: mBio. 2023 Jul 26;14(4):e01403-23. doi: 10.1128/mbio.01403-23 (PMC10470492; doi:10.1128/mbio.01403-23)

**FIGURE S2.** Representative modes of binding at the entrance channel of the L monomer of MexB. (**A**) SUB58 (blue, score: -10.6 kcal/mol). The representative docking pose of SUB58, with the 3-fluoro-4-(trifluoromethyl)benzyl group that lies on the hydrophobic part of the AP_L_, in a region delimited by L564, P669, V671, L674 and L861, while the polycationic moiety faces the periplasm. The polyamide linker allows the 2-azaniumyl-ethyl branches to engage stabilizing hydrogen bonds, anchoring the SUB58 to the pocket entrance (**B**) EPI18 (orange, score: -11.9 kcal/mol) assumes a configuration almost superimposable with that of SUB58 in terms of general orientation of the *p*-trifluoromethylphenyl (inward) and the 2-azaniumyl-ethyl groups (outward). (**C**) EPI-S32 (gray, score: -11.6 kcal/mol) forms a hydrogen bond with E829, which seems to contribute to the stabilization of the compound at the AP_L_. (**D**) AVD108 (yellow, score: -12.9 kcal/mol) presents an interaction pattern like that of the other compounds (with the polar amines pointing towards the periplasm and the aromatic rings facing the inner portion of the pocket). Interactions with residues are highlighted as dotted lines.

**(A)** **(B)**

**
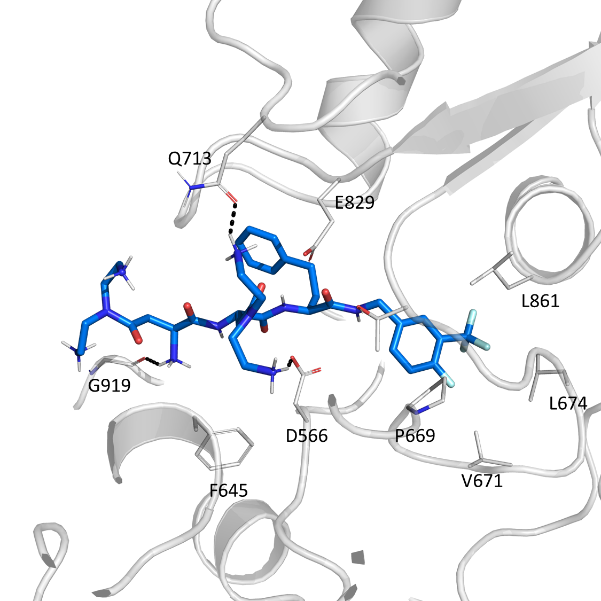

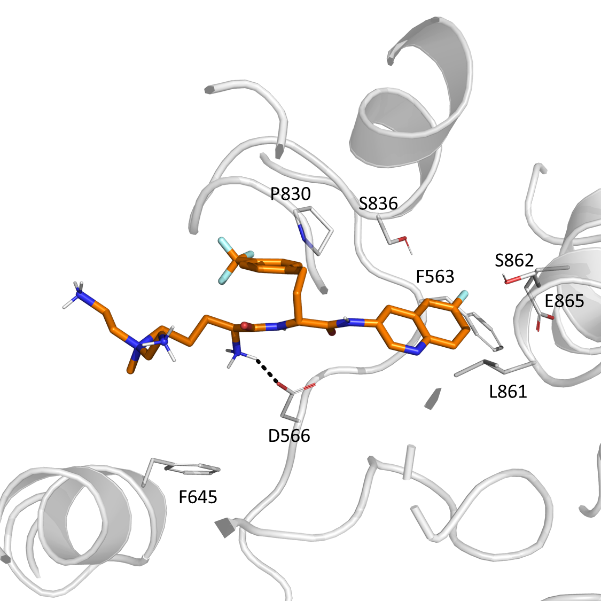
**

**(C)** **(D)**


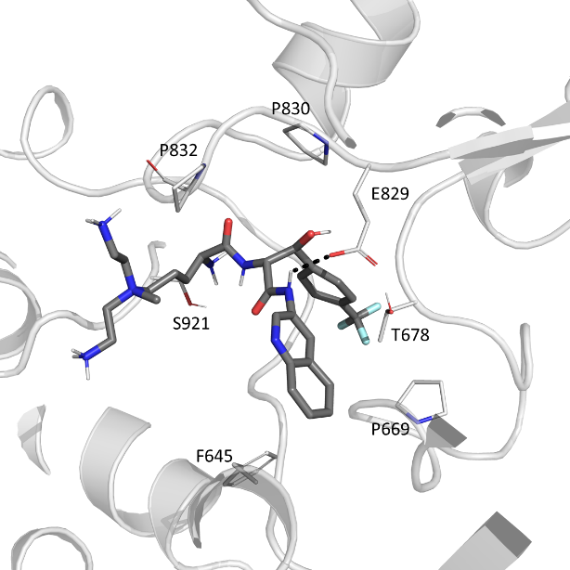

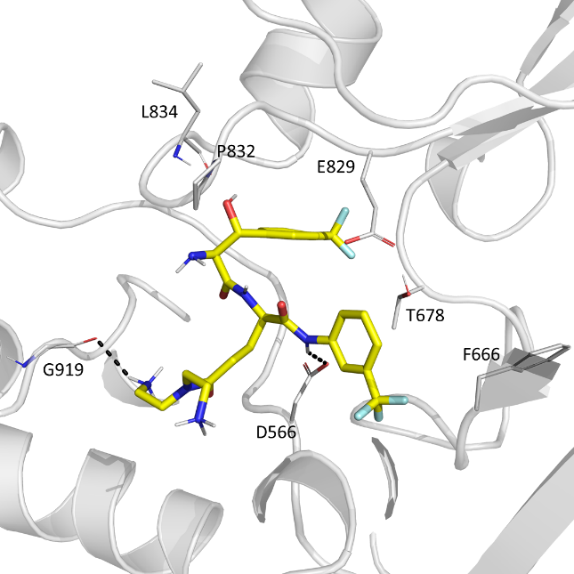

Supplement: Fig. S2 — Representative modes of binding at the entrance channel of the L monomer of MexB. [file mbio.01403-23-s0003.docx]
