# Supplementary material for: Molecular determinants of avoidance and inhibition of Pseudomonas aeruginosa MexB efflux pump
Source: mBio. 2023 Jul 26;14(4):e01403-23. doi: 10.1128/mbio.01403-23 (PMC10470492; doi:10.1128/mbio.01403-23)

**FIGURE S3.** Same as Figure 2 for the representative binding modes at the T monomer of MexB. (**A**) SUB58 (blue, score -13.6 kcal/mol) elicits both hydrophobic (F610, F617) and polar (Q46, N718, E825) contacts. Although the 3-fluoro-4-(trifluoromethyl)benzyl group of SUB58 lies in the hydrophobic trap, no *π-π* interactions were found.). (**B**) EPI18 (orange, score -13.7 kcal/mol) is mainly involved in hydrophobic interactions (F136, F573, F615, F617 belonging to or near the hydrophobic trap), and in one hydrogen bond with T91. (**C**) EPI-S32 (gray, score: -13.8 kcal/mol) establishes different interactions in the DP_T_. The specific chirality constrains the compound in terms of interaction with the hydrophobic trap, where the quinoline and the *p*-trifluoromethylphenyl contact several phenylalanine residues, but only the former establishes clear π-π stacking with F628 and F615, while the latter point towards V139, P326 and M630. A hydrogen bond is present, involving the cationic group and F617. Interactions with residues are highlighted as dotted lines.

**(A)** **(B)**


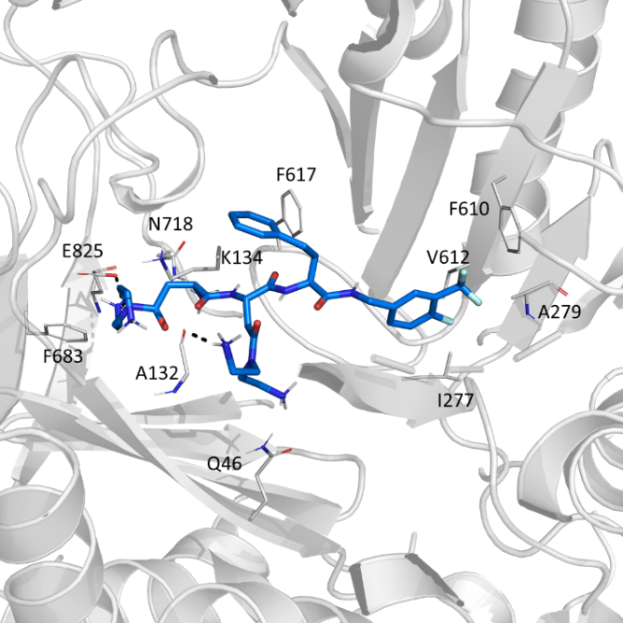

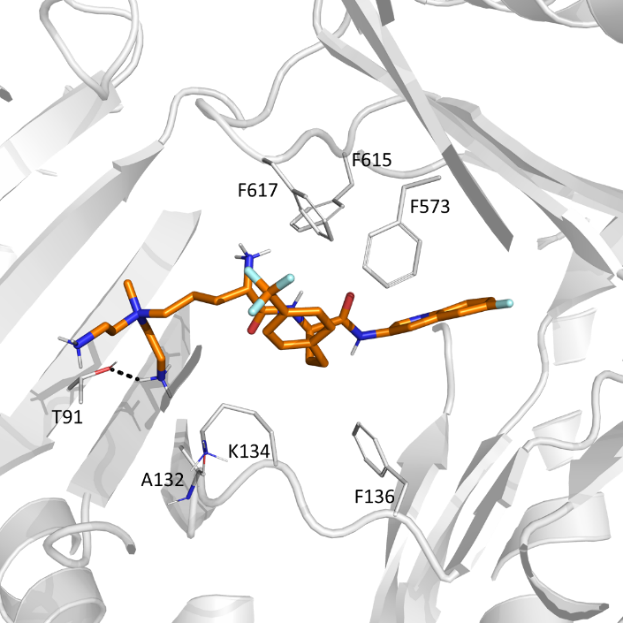


**(C)**


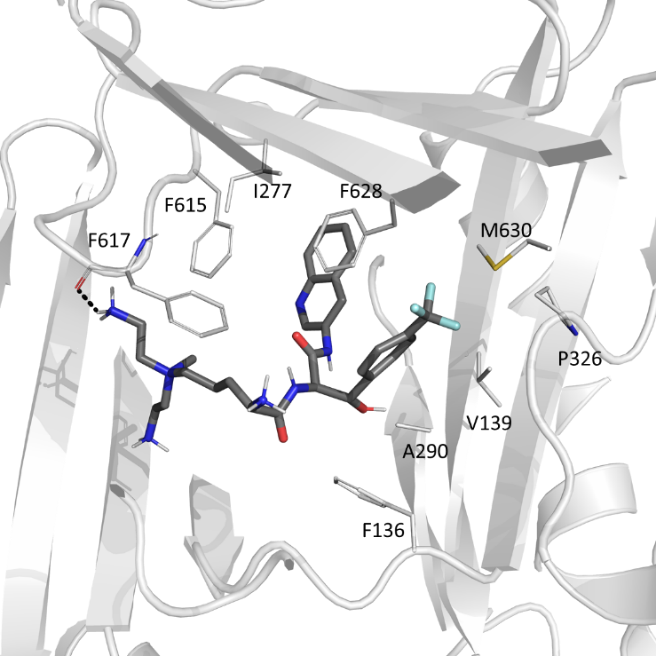

Supplement: Fig. S3 — The representative binding modes at the T monomer of MexB. [file mbio.01403-23-s0004.docx]
