# Supplementary material for: Molecular determinants of avoidance and inhibition of Pseudomonas aeruginosa MexB efflux pump
Source: mBio. 2023 Jul 26;14(4):e01403-23. doi: 10.1128/mbio.01403-23 (PMC10470492; doi:10.1128/mbio.01403-23)
Supplement: Fig. S4 — Mean RMSD values. [file mbio.01403-23-s0005.docx]

**FIGURE S4.** Mean RMSD values (Å) with the corresponding standard deviations of the ten MD replicas, performed at the AP_L_ (left) and DP_T_ (right). (**A**) SUB58, (**B**) EPI18, (**C**) EPI-S32 and (**D**) AVD108.

| AP_L_  (**A**) SUB58  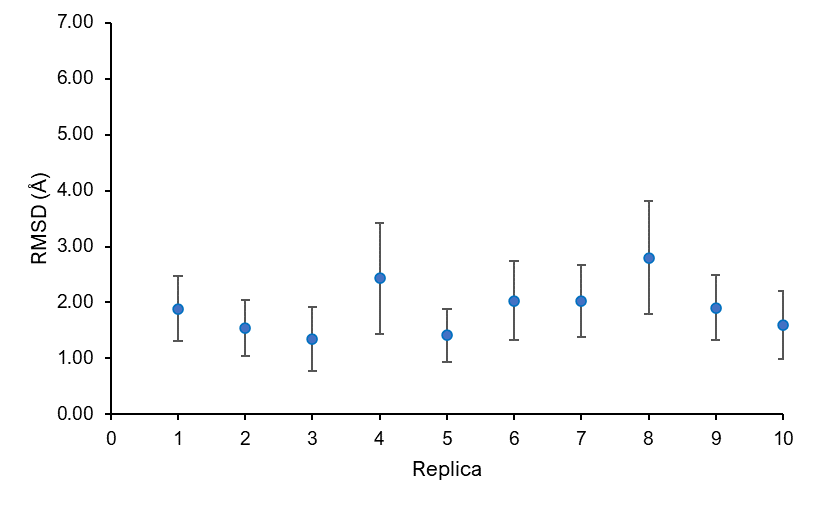 | DP_T_  _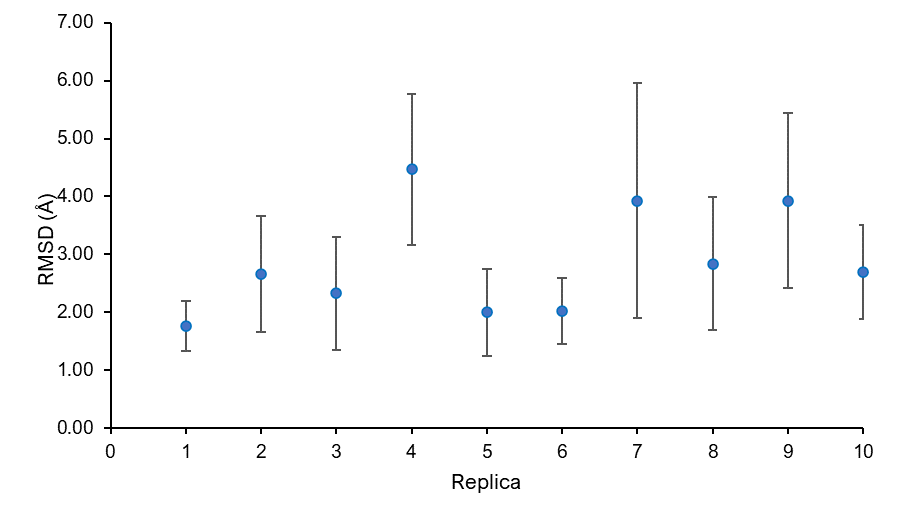_ |
| --- | --- |
| (**B**) EPI18  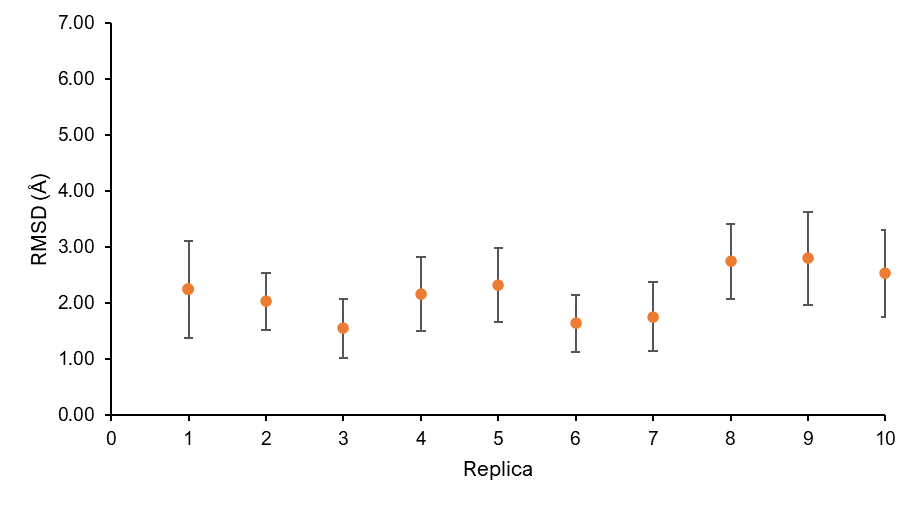 | 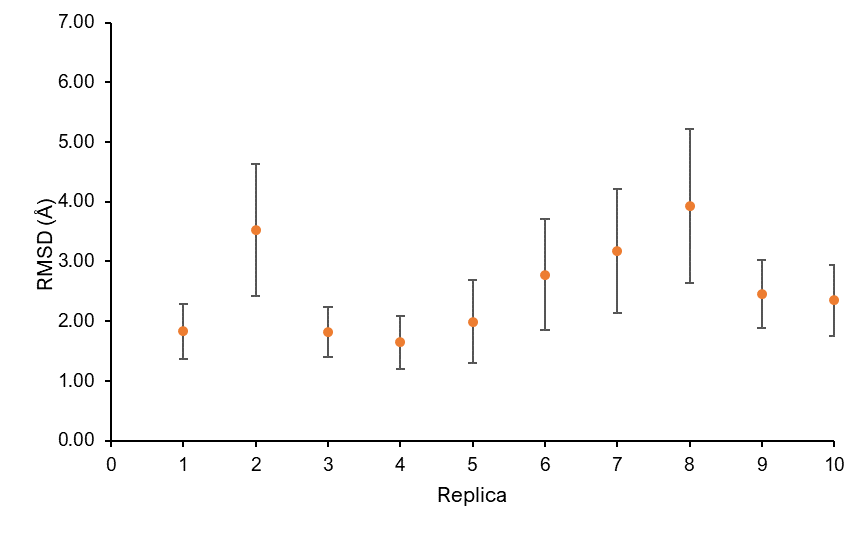 |
| (**C**) EPI-S32  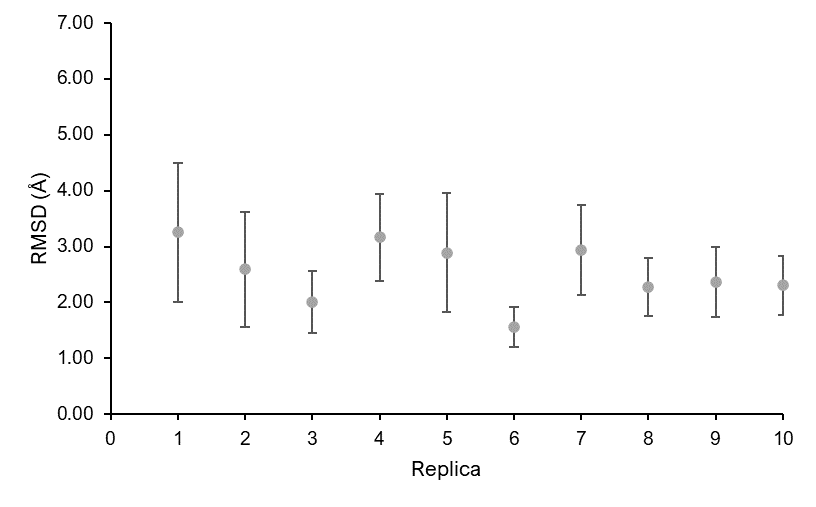 | 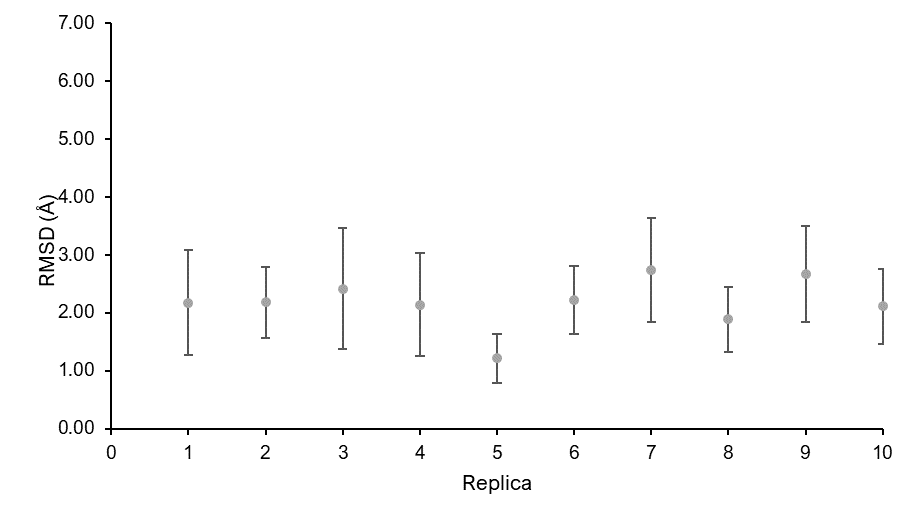 |
| (**D**) AVD108  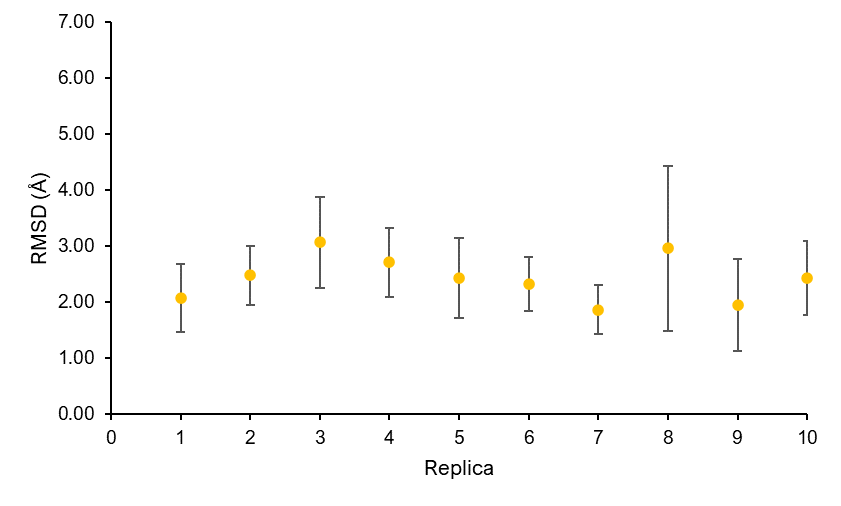 |  |
