# Supplementary material for: Molecular determinants of avoidance and inhibition of Pseudomonas aeruginosa MexB efflux pump
Source: mBio. 2023 Jul 26;14(4):e01403-23. doi: 10.1128/mbio.01403-23 (PMC10470492; doi:10.1128/mbio.01403-23)
Supplement: Fig. S5 — Pharmacophore groups of Rempex compounds used in contact H-bond water-mediated bridge analyses. [file mbio.01403-23-s0006.docx]

**FIGURE S5.** Pharmacophore groups of Rempex compounds used in contact H-bond water-mediated bridge analyses. (**A**) SUB58, (**B**) EPI18, (**C**) EPI-S32, and (**D**) AVD108. Spheres are colored according to the type of pharmacophore assigned to the functional group: purple for aromatic, cyan for H-donor, blue for H-donor and positively charged, orange for H-acceptor, and green for halogen.

| **(A)**  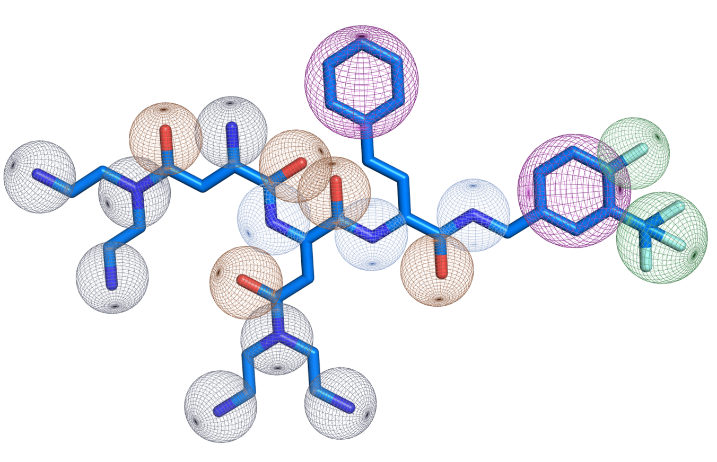 | **(B)**  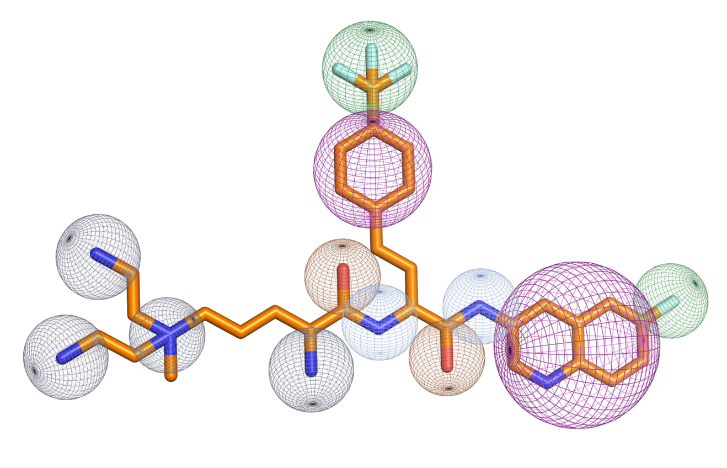 |
| --- | --- |
| **(C)**  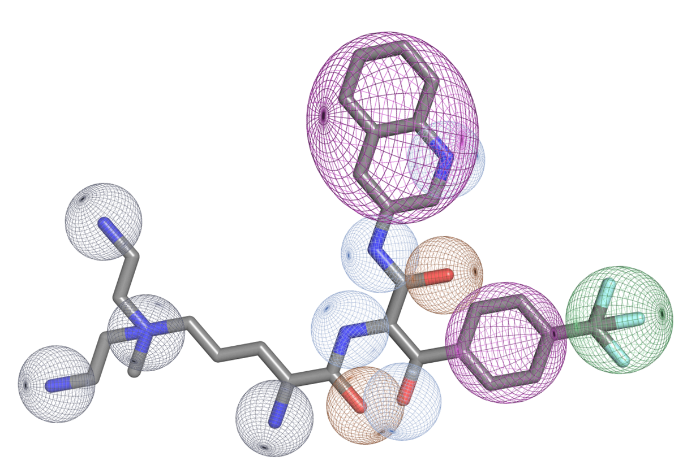 | **(D)**  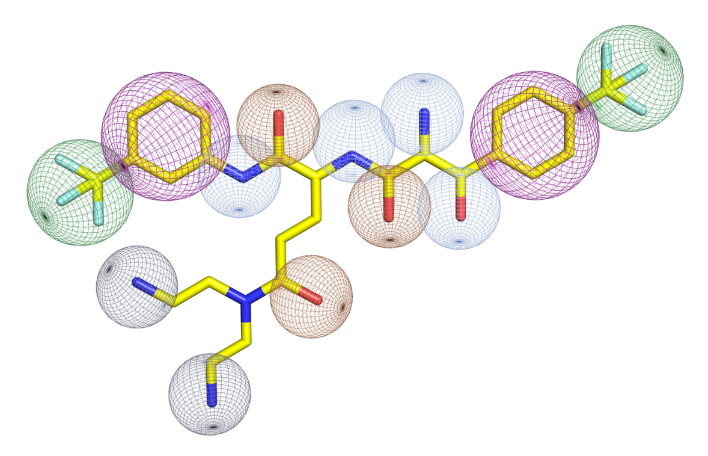 |
