# Supplementary material for: Molecular determinants of avoidance and inhibition of Pseudomonas aeruginosa MexB efflux pump
Source: mBio. 2023 Jul 26;14(4):e01403-23. doi: 10.1128/mbio.01403-23 (PMC10470492; doi:10.1128/mbio.01403-23)
Supplement: Fig. S6 — Relative levels of expression of MexB variants. [file mbio.01403-23-s0007.docx]

**Figure S6**. Relative levels of expression of MexB variants. Membrane fractions were isolated by ultracentrifugation and proteins were resolved using 10% SDS-PAGE. MexB variants were visualized by immunoblotting with monoclonal anti-His tag antibody (Sigma) and the intensity of the bands was measured by densitometry. The expression of MexB variants is shown as a percent of the expression of MexB WT loaded and analyzed on the same immunoblots. Error bars are SE (n=2).
