# Supplementary material for: Molecular determinants of avoidance and inhibition of Pseudomonas aeruginosa MexB efflux pump
Source: mBio. 2023 Jul 26;14(4):e01403-23. doi: 10.1128/mbio.01403-23 (PMC10470492; doi:10.1128/mbio.01403-23)

**Figure S7.** Growth inhibition of PA2859(Pore) cells producing the indicated MexB variants by EPI-S32. Cells carrying MexB and E81A were grown in LB broth supplemented with 32 mg/L of NOV and increasing concentrations of EPI-S32. Cells carrying E81C and V671A were grown at 16 mg/L of NOV and increasing concentrations of EPI-S32. Optical density was measured after 24 hrs of incubation and plotted as a function of EPI-S32 concentration. Error bars SD (n=2).


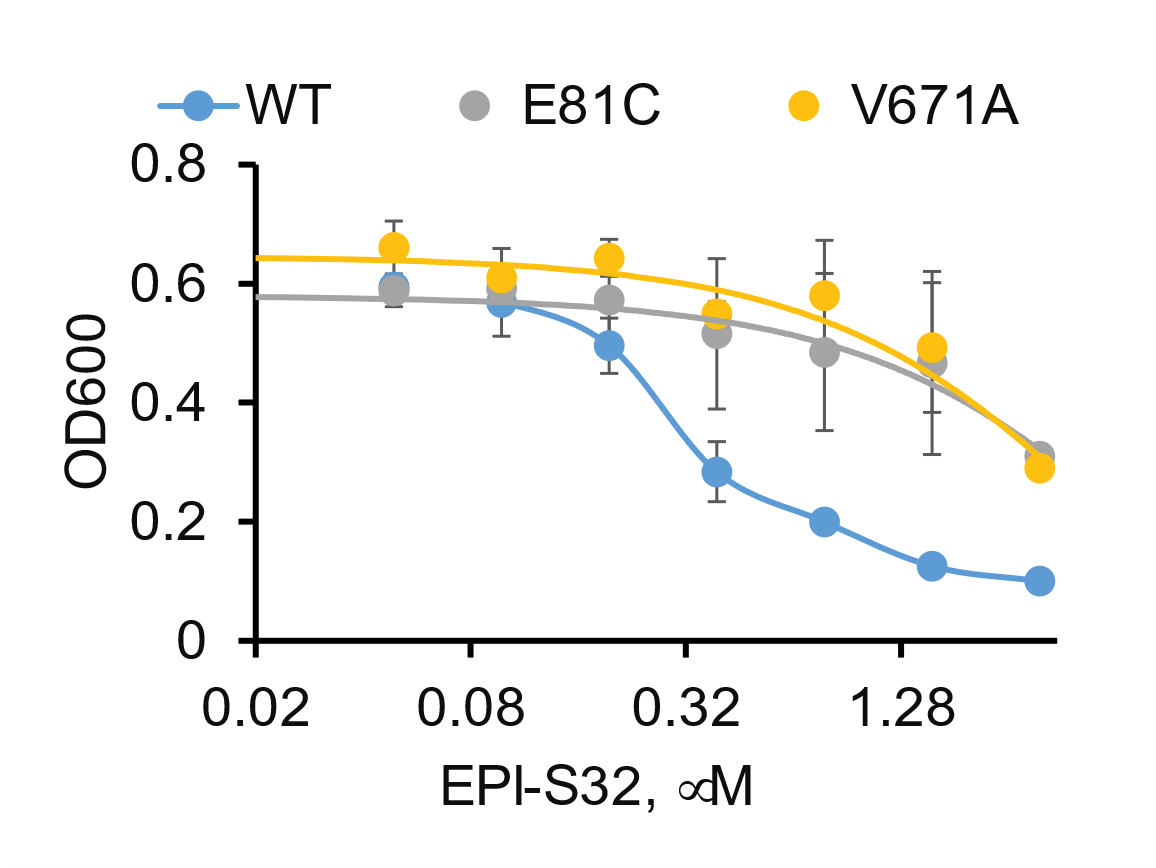

Supplement: Fig. S7 — Growth inhibition of PA2859(Pore) cells producing the indicated MexB variants by EPI-S32. [file mbio.01403-23-s0008.docx]
